# Detection of Herpes Simplex and Varicella-Zoster Virus in Clinical Specimens by Multiplex Real-Time PCR and Melting Curve Analysis

**DOI:** 10.1155/2014/261947

**Published:** 2014-04-16

**Authors:** Yun Ji Hong, Mi Suk Lim, Sang Mee Hwang, Taek Soo Kim, Kyoung Un Park, Junghan Song, Eui Chong Kim

**Affiliations:** ^1^Department of Laboratory Medicine, Seoul National University College of Medicine, Seoul 110-744, Republic of Korea; ^2^Department of Laboratory Medicine, Seoul National University Bundang Hospital, 173-82 Gumiro, Bundanggu, Seongnam, Gyeonggido 463-707, Republic of Korea

## Abstract

Herpes simplex viruses types 1 and 2 (HSV-1 and HSV-2), and varicella-zoster virus (VZV) are common agents resulting in various forms of clinical manifestation from skin vesicle to disseminated viral infection. The aim of the present study was to develop a real-time PCR and melting curve analysis which detect and differentiate HSV-1, HSV-2, and VZV, to compare with PCR-RFLP using clinical specimens, and to introduce the 4-year experience in the clinical laboratory. Three pairs of primers for HSV-1, HSV-2, and VZV were designed. Primers for human endogenous retrovirus-3 (HERV-3), an internal control, were adopted. A hundred selected specimens and many clinical specimens were tested for methods comparison and assay validation. Increased sensitivity and specificity were obtained from real-time PCR. In review of results of clinical specimens submitted to clinical laboratory, a total of 46 of 3,513 specimens were positive in cerebrospinal fluids, blood, skin vesicles, genital swabs, aqueous humor, and ear discharge. Thus, this method could be a rapid and accurate alternative to virus culture and other molecular tests for detection and typing of HSV-1, HSV-2, and VZV.

## 1. Introduction

Alphaherpesvirinae, a subfamily of Herpesviridae, is a common causative agent of human virus infection and includes herpes simplex viruses types 1 and 2 (HSV-1 and HSV-2) and varicella-zoster virus (VZV). Although they are famous for resulting vesicles in skin, the clinical manifestations which involve other areas than cutaneous area are more concerned. Especially in visceral organs and central nervous system (CNS) involvement, the suspicion of the infection is difficult, let alone the diagnosis, because symptoms and signs are ambiguous [1].

In clinical laboratory, conventional methods for detecting HSV and VSV such as cell culture or direct immunofluorescent assay (DFA) have limitations such as slowness, insensitivity, and nonstandardization in interpretation [2]. Moreover, the illness differences in severity and antiviral regimens according to viral species have been ascertained which requires more time, labor, and cost in traditional methods [3, 4].

Polymerase chain reaction- (PCR-) based techniques, particularly in CNS infection, have replaced the gold standard for the diagnosis of HSV-1, HSV-2, and VZV because cerebrospinal fluid stays positive for up to 1 week in the infection [3]. Various PCR-based methods have been introduced to the clinical laboratory, for example, conventional PCR, nested PCR, and PCR-restriction fragment length polymorphism (RFLP). Also, real-time PCR has been established as an easily available assay of microbiology laboratory recently, with the advantage of rapidity and low contamination rate. The aim of the present study was to develop a real-time PCR and melting curve analysis which detect and differentiate HSV-1, HSV-2, and VZV with LightCycler SYBR Green PCR, to compare with PCR-RFLP using clinical specimens, and to introduce the 4-year experience in the clinical laboratory.

## 2. Materials and Methods

### 2.1. Patients and Specimens

Specimens used in this study were classified into two groups: 100 specimens including already known as positives for clinical validation and clinical specimens obtained from patients with signs and symptoms suggestive of HSV or VZV infection for diagnosis. The 100 specimens consisted of 79 cerebrospinal fluids (CSF), 20 vesicle swabs, and 1 plasma sample.

### 2.2. Shell Vial Culture and Typing

Human lung fibroblast (MRC-5 cells) cultured in modified Eagle's medium (MEM) containing 10% fetal bovine serum, gentamicin, vancomycin, and nystatin was used for the preparation of cell monolayer. Cells were inoculated with 100 *μ*L of filtered clinical specimens and centrifuged at 700 ×g for 40 min. Thereafter, 1 mL of culture medium was added to the shell vials and the cultures were incubated at 37°C. Virus typing was performed on days 2, 4, and 6 after inoculation using monoclonal antibodies specific for HSV-1, HSV-2, or VZV (Light Diagnostics HSV 1/2 DFA and Light Diagnostics Varicella-Zoster DFA; Chemicon International, Temecula, CA, USA).

### 2.3. Nucleic Acid Extraction

60 *μ*L of extracted material was obtained from 140 *μ*L of clinical specimen by QIAamp Viral RNA Mini Kit (Qiagen, Valencia, CA, USA) according to the manufacturer's instructions. PCR-RFLP and real-time PCR and melting curve analysis were performed with the identical nucleic acid.

### 2.4. Polymerase Chain Reaction-Restriction Fragment Length Polymorphism (PCR-RFLP)

Genes encoding DNA polymerase of HSV and thymidine kinase of VZV were amplified for PCR-RFLP [5, 6]. After treating the amplified nucleic acid with* Sma *I and* Bam*H I for HSV-1 and HSV-2 and* Sma* I only for VZV, the PCR products were visualized using 2% agarose gel electrophoresis to detect the fragmentation.

### 2.5. Real-Time PCR and Melting Curve Analysis

The primers used in amplification and the gene targets are shown in Table 1. All primers except a pair for internal control were designed using LightCycler probe design software 2.0 (Roche, Penzberg, Germany). Human endogenous retrovirus-3 (HERV-3)* env *gene was also used to monitor false-positive results due to extraction failure or presence of inhibitors [7, 8]. The reaction mixture (20 *μ*L), containing 3.0 *μ*L of extracted nucleic acid, 1 × LightCycler FastStart DNA Master SYBR Green I (Roche), 3 mM MgCl_2_, 0.3 *μ*M of primers for HERV-3, and each of the primers for the detection (1.0 *μ*M of primers for VZV or 0.3 *μ*M of each primer for HSV), was denatured initially for 10 min at 95°C and was then treated for 5 sec at 95°C, 3 sec at 65°C, and 10 sec at 72°C for 45 cycles with LightCycler 2.0 (Roche). The program for analytic melting was followed by an increase in temperature to 99°C from 65°C with a 0.1°C/s ramp rate.

### 2.6. Standard Materials for Evaluation of Analytic Sensitivity of Real-Time PCR

Nucleic acid was amplified by the same primers used in multiplex real-time PCR and inserted into pTA2 vector using Target Cloning Kit (Toyoko, Osaka, Japan). Of the transformed, 3 colonies were selected for culture in Luria-Bertani media overnight, and then the plasmid DNA was extracted by GeneAll Exprep Plasmid SV Mini Kit (GeneAll Biotechnology, Seoul, Korea). With the restriction enzymes,* Hin*d III and* Bam*H I, transformation was confirmed and plasmid copy number was calculated. Creating standard curves of 10^2^ copies to 10^6^ copies per reaction by 10-fold distilled water dilution, the sensitivity of real-time PCR and melting curve analysis was determined.

## 3. Results

### 3.1. Validation of Multiplex Real-Time PCR and Melting Curve Analysis

**Figure 1:**
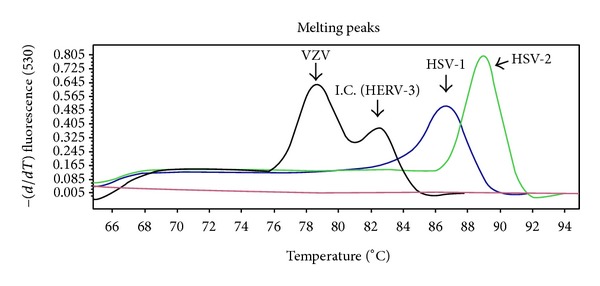
Differentiation of HSV-1, HSV-2, and VZV by multiplex real-time PCR-melting curve analysis. Melting peaks of HSV-1, HSV-2, and VZV positive samples by melting curve analysis. The HSV-1 amplicon has a melting temperature around 86.89°C, HSV-2 around 89.08°C, and VZV around 78.58°C, and internal control has a melting temperature around 82.41°C. HSV: herpes simplex virus; VZV: varicella-zoster virus; HERV-3: human endogenous retrovirus; I.C.: internal control.

In melting curve analysis, HSV-1, HSV-2, and VZV were distinguishable from each other, as well as HERV-3, the internal control (Figure 1). The melting curve of HSV-1, HSV-2, VZV, and HERV-3 showed the average melting temperature (Tm) to be 87.04°C, 89.32°C, 78.60°C, and 82.00°C, respectively (range: 86.84–87.16°C, 89.04–89.66°C, 77.36–79.20°C, and 81.09–82.89°C). In addition, the limits of detection were 1 copy, 10 copies, and 1 copy for HSV-1, HSV-2, and VZV, respectively (data not shown).

### 3.2. Comparison of Virus Culture, PCR-RFLP, and Real-Time PCR and Melting Curve Analysis in the Clinical Specimens

Of the total 100 clinical specimens, 9 (9%) were positive in multiplex real-time PCR, 8 (8%) in PCR-RFLP, and 4 (4%) in culture for HSV-1. Furthermore, 4 specimens were found positive only by molecular test and 1 specimen (CSF) was positive by real-time PCR solely. In HSV-2, 6 (6%) were positive in the respective molecular assays, and 4 (4%) were positive in culture. The assay of VZV was positive by real-time PCR and melting curve analysis in 19 (19%) specimens and by PCR-RFLP in 13 (13%) specimens. Of them, 6 specimens (5 CSF and 1 vesicle swab) were positive only by real-time PCR and melting curve analysis. None of the VZV cultures was positive in this study (Table 2).

### 3.3. Application of the Real-Time PCR and Melting Curve Analysis on Clinical Specimens

Between January 2008 and May 2012, more than 2,600 specimens were requested for detection of HSV-1, HSV-2, or VZV to Seoul National University Bundang Hospital, the tertiary referral center. The specimens types and number of tested specimens are shown in Table 3. Of 2,642 specimens for HSV-1 or HSV-2, 3 CSF, 1 blood sample, and 1 skin vesicle were positive for HSV-1 and so were 4 CSF, 3 skin vesicles, and 3 genital swabs for HSV-2. Of 871 specimens submitted for VZV, 30 (23 CSF, 2 blood samples, 2 skin vesicles, 2 aqueous humor, and 1 ear discharge) were positive.

Of the 30 virus-positive cases from 28 patients, 17 (60.7%) occurred in male patients and 11 (39.3%) occurred in female patients. The mean age was 54.2 years (range: 16–82 years; median: 59 years). Age, clinical diagnosis, and immunologic status of patients with virus-positive CSF are shown in Table 4. All 3 cases of HSV-1 positive patients resented with mental status changes. HSV-2 infections were diagnosed with meningitis presenting headache and fever in all immunocompetent patients. In cases of VZV, meningitis, myelitis, encephalitis, Ramsay Hunt syndrome, and disseminated viral infection were manifested as an infection.

## 4. Conclusion

HSV-1, HSV-2, and VZV, highly contagious viruses which belong to Alphaherpesvirinae, cause various forms of clinical manifestation from skin vesicles to disseminated viral infection [9]. Laboratory detection of these virus infections as well as clinical symptoms or signs is also important for diagnosis, as prompt antiviral therapy improves morbidity and mortality [10]. Many types of diagnostic methods for virus detection have been used in clinical laboratory so far; however, virus isolation in cell culture, known as classical “gold standard,” has several limitations in turnaround time, manual labor, and lack of sensitivity. Our results on real-time PCR and melting curve analysis in HSV-1, HSV-2, and VZV clearly indicated increased diagnostic sensitivity compared to shell vial culture. Especially, the detection rates of VZV were markedly improved in the newly developed assay, which correlates with previous report by others [11].

In comparison with PCR-RFLP, real-time PCR and melting curve analysis demonstrated a modest increased sensitivity. General limitation has to be taken into consideration nevertheless: long turnaround time as ever due to cumbersome post-PCR processing and nonnegligible contamination chances transferring amplicons onto agarose gel [12]. In this study, real-time PCR using SYBR green dye was chosen since it does not require setting new probes. SYBR green chemistry intercalates double-stranded DNA, amplifies fluorescent signals a thousandfold as PCR progresses, and finally detects the presence of target DNA. It is suitable for clinical molecular laboratories because it is employable for other PCR items in addition to its inexpensiveness compared with hydrolysis probe. The SYBR green chemistry has limits of nonspecific reaction to double-stranded DNA and possibility of false-positive results; thus it needs optimization in the quantification in assay [13]. However, real-time PCR used in this study, by implementation of melting curve analysis, increased reliability of results and solved the problem of SYBR Green I, which binds to nonspecific double-stranded DNA [14].

HERV-3, relatively well characterized among endogenous retroviruses, is reported to be present in the human genome at a single genomic locus. Because of its properties, HERV-3 is often used to measure both DNA quality and quantity and to monitor PCR inhibitors [7]. There are several reports that undiluted and untreated specimens have a much greater incidence of inhibition [8, 15]. More than 11 types of specimens were submitted to the clinical laboratory and little is known about presence of inhibitors in different kinds of specimens, especially in the rarely requested items. As multiplex real-time PCR assays for HSV-1, HSV-2, and HERV-2 (VZV and HERV for another tube) are devised in this study, it is expected to detect PCR inhibitors.

Laboratory diagnosis and typing of herpesviruses are important for some complications such as meningitis and encephalitis. While HSV-2 is related to recurrent meningitis and meningoencephalitis in immunocompromised patients, HSV-1 is generally known to be the most common cause of sporadic encephalitis [16]. Although positive cases are rare and unfeasible to generalize in the present study, the findings of CSF positive patients were concordant with others. With long-term extensive study, it is expected to define patients' demographics and epidemiology of HSV-1, HSV-2, and VZV.

In summary, the newly developed real-time PCR and melting curve analysis have increased sensitivity and shortened turnaround time in clinical samples. The Accurate and less labor intensive molecular assay in clinical laboratory may help the rapid diagnosis and prompt treatment for patients.

## Figures and Tables

**Table 1:** Primer sequences used and product sizes of each target genes for HSV-1, HSV-2, VZV, and HERV-3.

Virus	Gene target	Primer sequences (5′→3′)	Amplicon (bp)	Reference
HSV-1	*gpD *	Forward primer: GGTCTCTTTTGTGTGGTGC	84	This study
		Reverse primer: GCCCACTATGACGACAAAC		This study

HSV-2	*gpG *	Forward primer: TACGCTCTCGTAAATGCTTC	120	This study
		Reverse primer: GCCCACCTCTACCCACAA		This study

VZV	*ORF4 *	Forward primer: GCCCATGAATCACCCTC	79	This study
		Reverse primer: ACTCGGTACGCCATTTAG		This study

HERV-3	*envelope *	Forward primer: CATGGGAAGCAAGGGAACTAATG	135	Yuan et al., 2001 [7]
		Reverse primer: CCCAGCGAGCAATACAGAATTT		Yuan et al., 2001 [7]

HSV: herpes simplex virus; VZV: varicella-zoster virus; HERV-3: human endogenous retrovirus; *gpD*: glycoprotein D; *gpG*: glycoprotein G; *ORF4*: open reading frame 4.

**Table 2:** Comparison of real-time PCR, PCR-RFLP, and virus culture for the detection of HSV-1, HSV-2, and VZV during the validation phase.

Virus	Real-time PCR		PCR-RFLP		Virus culture	
	Positive	Negative	Positive	Negative	Positive	Negative
HSV-1	9	91	8	92	4	96
HSV-2	6	94	6	94	4	96
VZV	19	81	13	87	0	100

HSV: herpes simplex virus; VZV: varicella-zoster virus.

**Table 3:** Summary of specimen types and numbers positive for HSV-1, HSV-2, and VZV.

Specimen type	Number tested		Number positive		
	HSV-1 and HSV-2	VZV	HSV-1	HSV-2	VZV
CSF	2,495	755	3	4	23
Blood					
Serum	62	68	0	0	1
Plasma	15	16	1	0	1
Skin vesicle	16	13	1	3	2
Genital swab	21	0	0	3	0
Oral swab	4	2	0	0	0
Eye					
Corneal scraping	11	0	0	0	0
Vitreous fluid	8	8	0	0	0
Aqueous humor	3	5	0	0	2
Amniotic fluid	5	1	0	0	0
Urine	1	0	0	0	0
BAL	0	2	0	0	0
Ear discharge	0	1	0	0	1
Tissue (skin)	1	0	0	0	0

Total	2,642	871	5	10	30

HSV: herpes simplex virus; VZV: varicella-zoster virus; CSF: cerebrospinal fluid; BAL: bronchoalveolar lavage.

**Table 4:** Clinical and laboratory findings of the patients positive for the presence of HSV or VZV DNA in CSF by PCR.

Virus detected	Number of positive samples	Age at diagnosis (number of patients)	Clinical diagnosis (number of patients)	Number of immunocompromised patients*/number of immunocompetent patients
HSV-1	3	31–50 (1)	Encephalitis (1)	0/1
		>50 (2)	Encephalitis (2)	1/1

HSV-2	4	31–50 (2)	Meningitis (2)	0/2
		>50 (1)	Meningitis (1)	0/1

VZV	23	10–30 (3)	Meningitis (3)	0/3
		31–50 (7)	Meningitis (6)	1/5
			Myelitis (1)	0/1
		>50 (12)	Encephalitis (6)	4/2
			Meningitis (4)	1/3
			Disseminated viral infection (1)	1/0
			Ramsay Hunt syndrome (1)	0/1

*Immunocompromised patients include patients who were diagnosed with hematologic malignancy (acute myeloid leukemia, diffuse large B-cell lymphoma, and essential thrombocythemia), solid tumor (breast cancer and cervical cancer), rheumatoid arthritis treated with methotrexate, and diabetes mellitus.

HSV: herpes simplex virus; VZV: varicella-zoster virus.

## References

[1] Olmez D, Boz A, Erkan N (2009). Varicella zoster infection: a rare cause of abdominal pain mimicking acute abdomen. *Journal of Clinical Medicine Research*.

[2] Gleaves CA, Rice DH, Bindra R (1989). Evaluation of a HSV specific monoclonal antibody reagent for laboratory diagnosis of herpes simplex virus infection. *Diagnostic Microbiology and Infectious Disease*.

[3] Whitley RJ, Roizman B (2001). Herpes simplex virus infections. *The Lancet*.

[4] Sauerbrei A, Wutzler P (2007). Herpes simplex and varicella-zoster virus infections during pregnancy: current concepts of prevention, diagnosis and therapy. Part 2: Varicella-zoster virus infections. *Medical Microbiology and Immunology*.

[5] Kido S, Ozaki T, Asada H (1991). Detection of varicella-zoster virus (VZV) DNA in clinical samples from patients with VZV by the polymerase chain reaction. *Journal of Clinical Microbiology*.

[6] Rozenberg F, Lebon P (1991). Amplification and characterization of herpesvirus DNA in cerebrospinal fluid from patients with acute encephalitis. *Journal of Clinical Microbiology*.

[7] Yuan CC, Miley W, Waters D (2001). A quantification of human cells using an ERV-3 real time PCR assay. *Journal of Virological Methods*.

[8] Whiley DM, Mackay IM, Syrmis MW, Witt MJ, Sloots TP (2004). Detection and differentiation of herpes simplex virus types 1 and 2 by a duplex LightCycler PCR that incorporates an internal control PCR reaction. *Journal of Clinical Virology*.

[9] Dwyer DE, Cunningham AL (2002). 10: herpes simplex and varicella-zoster virus infections. *Medical Journal of Australia*.

[10] Long SS (2013). Delayed acyclovir therapy in neonates with herpes simplex virus infection is associated with an increased odds of death compared with early therapy. *Evidence-Based Medicine*.

[11] Espy MJ, Teo R, Ross TK (2000). Diagnosis of varicella-zoster virus infections in the clinical laboratory by LightCycler PCR. *Journal of Clinical Microbiology*.

[12] Heid CA, Stevens J, Livak KJ, Williams PM (1996). Real time quantitative PCR. *Genome Research*.

[13] Arya M, Shergill IS, Williamson M, Gommersall L, Arya N, Patel HRH (2005). Basic principles of real-time quantitative PCR. *Expert Review of Molecular Diagnostics*.

[14] Sibley CD, Peirano G, Church DL (2012). Molecular methods for pathogen and microbial community detection and characterization: current and potential application in diagnostic microbiology. *Infection, Genetics and Evolution*.

[15] Druce J, Catton M, Chibo D (2002). Utility of a multiplex PCR assay for detecting herpesvirus DNA in clinical samples. *Journal of Clinical Microbiology*.

[16] Kneen R, Michael BD, Menson E (2012). Management of suspected viral encephalitis in children-association of British Neurologists and British Paediatric Allergy, Immunology and Infection Group National Guidelines. *Journal of Infection*.

